# At the Society of Arts on Wednesday

**Published:** 1887-10-22

**Authors:** 


### At the Society of Arts on Wednesday.

By One who was There.

If any person required proof of the intelligence and foresight of present-day hospital officials and sick-nurses as a class, the Pension Fund meeting, held on October 12th, was an occasion to bring ample conviction to the mind. Without special effort on anybody's part, but simply by the steady work of The Hospitals Association, and the freely opened columns of The Hospital, a widespread interest has been created, which brought together in the large lecture-hall of the Society of Arts upwards of five hundred persons of all ranks in the social, medical, and philanthropic worlds. Alike to those who looked at the spectacle from the outside standpoint of an active philanthropy, and to those who were, if possible, still more deeply interested in the subject as vital to themselves, the occasion was one for mutual congratulation and profound thankfulness. The President, Sir Andrew Clark, Bart., whose constant and diligent interest in the Association and all its plans and operations is one of the surest guarantees of its stability and public value, entered into the spirit of the pension movement with all the ardour of a sincere philanthropist, and all the wisdom of a practical man of business. Mr. Burdett, whose enthusiasm it is impossible to subdue, witnessed what to him was a splendid promise of the successful completion of years of laborious thought, deep anxiety, and unwearying effort in a great and benevolent cause.

The meeting was perhaps the largest and most broadly representative of any philanthropic gathering in London in recent years. The vast audience was an index to the state of feeling among hospital officials, especially of the matron and nursing class. The comparatively brief period during which trained nursing has been in extensive operation has been quite sufficient to convince those who have had personal experience that it is an arduous calling, attended with many risks, and can only be carried out for a lifetime by the exercise of self-denial and wise attention to health. In many instances a periodical breakdown must be anticipated and provided against; and a term of service, shorter by several years than the common duration of working life, must in every case be reckoned upon. Even the least thoughtful among nurses are beginning to find this out, whilst the more intelligent, who undoubtedly constitute by far the larger proportion, are deeply sensible of its truth. No doubt the habit of looking very far ahead is regarded by some as a kind of distrust of Providence, and as making life more anxious than it need necessarily be: and equally no doubt many nurses will marry, or revert for various reasons to non-professional life. But when all deductions are made, there must still remain a arge number to whom nursing will be a life-long occupation, and who, if they do not provide for old age out of their professional earnings, cannot provide for it at all. To such as these it presents a life of arduous and self-denying toil, with poverty, charity, or a still more melancholy lot at the end of it. Such an ending to a life spent in the service of others cannot be contemplated without indignation; and it is clearly the interest of nurses, as it is the duty of those to whom they may minister in sickness, to take advantage of any opportunity which offers of making a threefold provision against sickness, disablement, and advancing years.

Mr. Burdett, who spoke for an hour and ten minutes to an audience whose interest never wavered for a moment, unfolded a scheme which included all these essential features. For the payment of twelve shillings and sixpence per annum a sick insurance can be obtained which will give the subscriber one pound a week for six months during temporary disablement, and ten shillings a week up to sixty years of age if the disability be permanent; whilst for the further sum of two pounds ten, and sixpence a week, a retiring pension of from thirteen to fifteen pounds a year can be secured, without taking into consideration added bonuses. But there is more than a probability that such sums will be available in bonuses as will increase the allowance to each pensioner to a minimum of twenty-five pounds per annum. In other words, for the small payment of one shilling and twopence a week, a sick, a permanent disablement, and a pension fund can be created, which will secure to those who join it, as already stated, one pound a week for six months during temporary sickness, ten shillings a week up to the age of sixty in case of permanent inability to work, and, with added bonuses, ten shillings a week as a retiring pension for the remainder of life.

The scheme as it is here stated is a model of simplicity; but the labour and thought involved before such a result has been arrived at can only be appreciated by those who have knowledge of actuarial work. Two eminent actuaries, the late Mr. Cornelius Walford and Mr. George King, of the Atlas Insurance Company, have each given a vast amount of time and calculation to its construction and the elaboration of its details, and they have had the advantage of the experience of Mr. Burdett, which in all hospital questions may be said to be unique.

Without added bonuses the scheme is one which will confer inestimable benefits upon the nursing profession: with such bonuses it will raise nursing to the level of the public services. So that, if it be carried out, it may be said of anyone who enters the profession through the regular channels, and subscribes to the Fund, that she is provided for during the whole of life so long as she preserves an honourable character.

A word remains to be said about bonuses. A capital sum of twenty thousand pounds, invested in sound securities, will legally qualify the Fund to give pensions to any required amount. Without such a capital sum it must be registered under the Friendly Societies Act; and no pension for either nurse, matron, or hospital secretary can in that case exceed fifty pounds a year. The twenty thousand pounds is therefore essential to the proper working of the scheme. Her Majesty the Queen, it is said, proposes to place seventy-five thousand pounds, the amount of the Women's Jubilee Offering, at the disposal of selected persons or institutions for the benefit of the nursing profession. It is an idea eminently worthy of the Queen, and will gladden the hearts of many toiling and patient women for generations to come. It is hoped that Her Majesty will consider the advantages the Pension Fund would confer upon nurses and all hospital officials, and graciously direct that twenty of the seventy-five thousand she purposes to give shall be appropriated in this way. Thus, not only would the Fund be enabled to give adequate annuities to the better paid class of subscribers, but would also have the means of largely increasing the pensions of those who, unaided, can by no means subscribe enough annually to secure for themselves a retiring income sufficient for their modest wants.

The meeting was sustained with unabated interest to a late hour, and finally, on the motion of Lord Sandhurst, seconded by Mr. Pietro Michelli, a resolution was passed empowering the Executive of The Hospitals Association to definitely formulate the scheme outlined by Mr. Burdett, and to transmit it to Sir Henry Ponsonby for Her Majesty's gracious consideration. A vote of thanks to Sir Andrew Clark for presiding, and to Mr. Burdett for his able and comprehensive exposition, brought to a close one of the most interesting and important meetings ever held in connection with the hospital movement.

